# scTrimClust: a fast approach to robust scRNA-seq analysis using trimmed cell clusters

**DOI:** 10.1093/bioadv/vbag082

**Published:** 2026-03-16

**Authors:** Sergej Ruff, Klaus Jung

**Affiliations:** Institute of Animal Genomics, University of Veterinary Medicine Hannover, Hannover, 30559, Germany; Institute of Animal Genomics, University of Veterinary Medicine Hannover, Hannover, 30559, Germany

## Abstract

**Motivation:**

Detection of marker genes and other downstream analyses in single-cell sequencing experiments very much rely on the results of unsupervised clustering of cells. However, in two-dimensional representation of clustering results, several cells appear as outliers or in the border area of a cluster suggesting that these cells may also be outliers in the high-dimensional data space and do not adequately represent a particular cell type.

**Results:**

We propose a novel and fast approach, scTrimClust, for identifying cells that may be interpreted of extreme specimens of their cell type. Identification is based on measuring each cell’s distance to its nearest neighbours in the high-dimensional gene expression space, and marking those as extreme having minimum neighbour distance above a defined quantile threshold for that cluster. We study in two data examples, how cells with non-representative expression profile can influence the results of the analysis. scTrimClust is also useful to compare the influence of other parameters of an scRNA-seq analysis, e.g. normalization or the clustering approach, on the results. We also provide a software implementation of scTrimClust.

**Availability and implementation:**

The scTrimClust approach is available in the R-package RepeatedHighDim (https://cran.r-project.org/web/packages/RepeatedHighDim/index.html).

## 1 Introduction

Single-cell RNA-sequencing (scRNA-seq) (Kolodziejczyk *et al.* 2015) has become an important method in systems biology to analyse cell-type specific gene expression and has been applied to research on a wide range of diseases. For example, Gonzáles-Silva *et al.* (2020) used scRNA-seq to unravel tumour heterogeneity, and Melms *et al.* (2021) described the cell-type specific transcriptome in the lung under COVID-19 infections.

After mapping and counting of sequencing reads per gene, a high-dimensional expression matrix X with *d* rows representing several thousands of genes and *n* columns representing several hundred or thousands of individual cells from the tissue sample is the starting point of further analysis. Still, the number of rows exceeds usually the number of columns.

One fundamental step in the data analysis of scRNA-seq experiments is clustering and identification of cell types which can be done by unsupervised clustering methods such as the *k*-nearest neighbour (kNN) clustering based on the dimension-reduced expression matrix X. Depending on the type of tissue that is under research, clustering results in several more frequent cell types with several hundred cells and several less frequent cell types with one hundred cells or fewer.

While clustering itself is mostly done in the space of a few principal components, the clustering results are usually visualized in a 2-dimensional t-distributed Stochastic Neighborhood Embedding (t-SNE) (Van der Maaten and Hinton 2008) or Uniform Manifold Approximation and Projection (UMAP) (McInnes *et al.* 2018) plot. The shape of the different cell clusters in these plots can be quite different with some clusters having a concave or extremely skewed shape and sometimes having clear outliers. This suggests that the membership of outlying cells or cells in the border area of a cluster might not perfectly represent the cell type. Although these cells can be interpreted simply as biological extremes which should be kept in the analysis, there is also a risk that these cells might be classified into the wrong cluster. Other reasons for cells to have an extreme expression profile compared to the core cells of the cluster may be that these are cells in transition states (Wang *et al.* 2024) or in a differentiation continuum. Anyhow, these cells can have a strong impact on the results of the whole analysis.

To downweigh the effect of extreme observations, trimmed estimators have been used in statistics since the 1980s (Huber and Ronchetti 2011). For example, the trimmed mean has been shown to be a robust estimator in the presence of extreme observations. In univariate or bivariate data, outliers or extreme observations can be identified by statistical tests or graphically, e.g. using box-and-whiskers-plots or bagplots (Kruppa and Jung 2017). Following the idea of trimmed estimators, we introduce here a novel approach for fast identification of outlying cells. Instead of relying on geometric properties of two-dimensional embeddings, our method quantifies how well each cell is embedded within its cluster in high-dimensional gene expression space by measuring distances to its nearest neighbours. Cells with unusually large minimum distances to neighbouring cells are interpreted as lying in sparsely populated or border regions of the cluster and are therefore considered extreme. Since cell cluster in a t-SNE or UMAP plot often show a skewed or concave shape, existing methods for outlier detection are not appropriate. Therefore, extreme cells are identified within each cluster directly in the high-dimensional normalized gene expression space based on their distance to nearest neighbouring cells.

The effect of extreme and near border cells can then be studied during the analysis. Given that the results of scRNA-seq data analysis is influenced by several parameter settings, our approach is very helpful to study the robustness of the whole analysis. For example, the choice of the normalisation method, of the number of principal components used during unsupervised clustering, and of gene filtering can impact the overall results. While robustness of a scRNA-seq analysis can also be studied using time-consuming bootstrap procedures, the novel approach presented here simply contrasts the whole dataset versus a reasonably trimmed dataset and is therefore much faster.

In this manuscript, we describe our novel approach for trimming sets of cell types based on the identification of extreme and near border cells. We demonstrate the practicability of our approach on two data examples: first the dataset of Peripheral Blood Mononuclear Cells (PBMC) that is also used in the tutorial of the Seurat R-package (Satija *et al.* 2015), second a COVID-19 dataset. We also provide a description of how to use our novel R-function that implement the scTrimClust approach.

## 2 Methods

### 2.1 Data examples: peripheral blood cells and whole blood samples

We evaluated our approach using two publicly available scRNA-seq datasets. The first one, named PBMC 3k, was generated from peripheral blood mononuclear cells, is featured in the Seurat guided tutorial and is accessible through the Seurat package or the 10× Genomics webpage (https://cf.10xgenomics.com/samples/cell/pbmc3k/pbmc3k_filtered_gene_bc_matrices.tar.gz). It includes 2700 PBMCs with 13 714 features from healthy human donors.

The second dataset, focusing on SARS-CoV-2 infection, contains whole blood samples depleted of red blood cells from seven COVID-19-positive patients (stratified into mild, moderate, and severe groups) and from three SARS-CoV-2-negative controls. For our analysis, we retained only the 5295 cells and 38 949 features from the 7 positive cases. This dataset, originally generated by Silvin *et al.* (2020), is accessible via the ArrayExpress Archive of Functional Genomics Data under accession number E-MTAB-9221 (https://www.ebi.ac.uk/biostudies/arrayexpress/studies/E-MTAB-9221).

### 2.2 Standard workflow of scRNA-seq analysis

Both datasets are first analysed using a standard workflow with no cells excluded from the analysis, following the analysis pipeline of the Seurat R-package (Satija *et al.* 2015). This pipeline consists of first filtering cells with extremely low (due to cell damage) or extremely high (due to non-dissolved cells) feature counts followed by normalisation. Thus, there is already some filtering regarding inappropriate data.

Thereafter, the dataset is further filtered by excluding genes with low variability over all cells as these genes may have little biological meaning. Typically, less than 5000 genes remain in the analysis. Next, linear dimension reduction by principal component analysis (PCA) is run and the unsupervised clustering is then performed on a couple of principal components. Results of the clustering are shown in t-SNE or UMAP plots. As final steps, marker genes per cluster are identified–usually the most strongly expressed genes per cluster.

Using the scTrimClust approach, we compare the results of marker gene detection under several different settings, i.e. with two normalisation methods (Seurat’s LogNormalize versus centered log ratio (CLR)), with two different sets of preselected genes (1000 versus 2000), and with two different numbers of principal components (5 versus 10) used for the clustering step.

### 2.3 scTrimClust workflow of scRNA-seq analysis

To identify extreme cells per cluster, we compute the *k*-nearest neighbour (kNN) distances within each cluster using the normalized gene expression space obtained in the standard analysis (see section 2.2). For this purpose, we apply a kNN search to all cells within a given cluster, identifying for each cell i a set of *k*-nearest neighbour cells. Next, we calculate the Euclidean distances $D_{j,i}$ between each cell i in this cluster and its j-th nearest neighbour in the high-dimensional expression space. The minimum distance of each cell to one of its nearest neighbours, $\min D_{i}=\min_{j=1,\ldots ,k}D_{j,i}$, is used to assess whether this cell belongs to the set of representative cells or rather to those located in sparsely populated regions at the periphery of the cluster. Results, for example a set of selected marker genes, can be compared by excluding a fraction of α of the most extreme cells, defined as those cells with min $D_{i}>Q_{1-\alpha }$ where $Q_{1-\alpha }$ denotes the (1-α) quantile of all min D values within each cluster.

### 2.4 Evaluation by set comparisons and breakdown point analysis

An essential goal of scRNA-seq is the selection of marker genes for individual cell types. Therefore, we compare the sets of selected markers under the different settings. For both datasets, COVID-19 and PBMC, we analysed how cells from the border areas influence the selection of marker genes under (i) different normalisation approaches, (ii) under different numbers of preselected genes, and (iii) under different numbers of principal components (PCs) used for unsupervised clustering.

Under each scenario and for each cell type we determined the set S_full_ of marker genes obtained with the full data and the set S_trim_ of marker genes obtained with the trimmed data. From these two sets, we derived the sets

S_1_ = S_full_\S_trim_ = {marker | marker ∈ S_full_  ∧ marker ∈ S_trim_},

S_2_ = S_full_  ∩ S_trim_ = {marker | marker ∈ S_full_  ∧ marker ∉ S_trim_}, and

S_3_ = S_trim_\S_full_ = {marker | marker ∈ S_trim_  ∧ marker ∉ S_full_},

i.e. the differences and the intersection between S_full_ and S_trim_. Based on the cardinalities |S_1_|, |S_2_|, and |S_3_|, i.e. the number of markers in each set S_1_, S_2_, and S_3_, we calculated the percent proportions

P_1_ = 100 · |S_1_|/ *m*,

P_2_ = 100 · |S_2_|/ *m*, and

P_3_ = 100 · |S_3_|/ *m*,

where *m* is the number of markers in the union of S_full_ and S_trim_, i.e. *m* = |S_full_  ∪ S_trim_|.

In the case of large proportion P_2_, a finding can be seen as robust since full and trimmed data yield a highly similar set of markers. If, however, P_1_ is large, it means that the selected markers with the full dataset highly rely on additional information from the cells in the border area. Finally, if P_3_ is large, it means that removing cells from the border area completely changes that set of selected markers.

In the area of robust statistics, estimators are often evaluated by their breakdown point, which is the proportion of extreme observations an estimator can cope with until it gives inaccurate results. Such a breakdown point analysis can also be done by comparing proportion P_1_ against P_2_ for different percentages of trimming. Hereby, the analyst can observe how robust a marker set is against the cells in the border area.

## 3 Results

### 3.1 Influence of cells from the border area on selection of marker genes

Here, we highlight exemplarily some scenarios with clear effects, further scenarios are presented as supplementary material.

**Figure 1:**
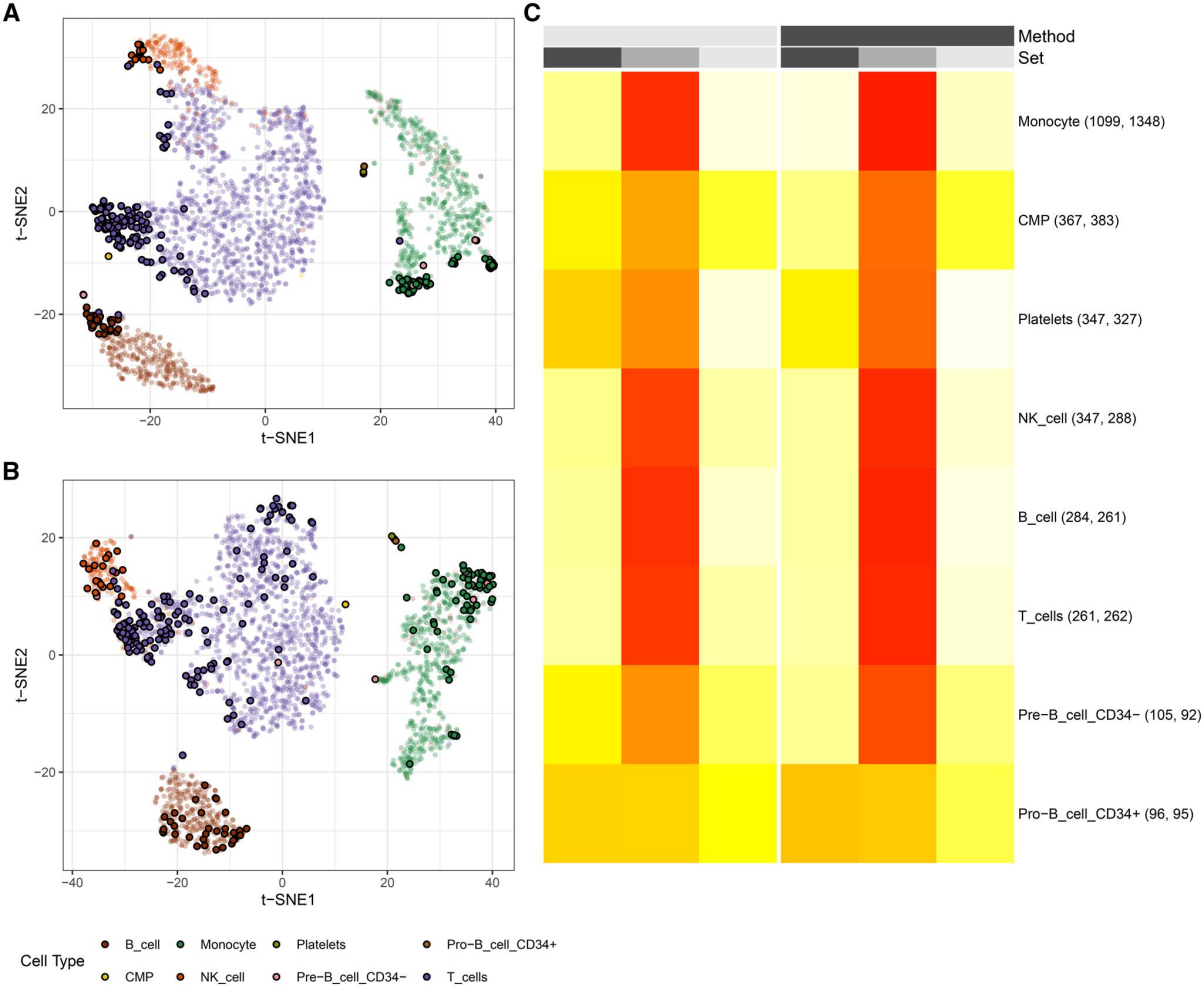
(A) t-distributed Stochastic Neighborhood Embedding (t-SNE) plot of the PBMC dataset after LogNormalize (LogNorm) normalisation. (B) t-SNE plot of the PBMC dataset after centered log ratio (CLR) normalisation. Both computed with 10 principal components and 2000 variable features. Cells identified as outliers (top 10% based on intracluster *k*-nearest neighbour distances) are highlighted with black outline. (C) Heatmap comparing results when using either LogNorm or CLR normalisation. Columns corresponding to LogNorm are shown in black, whereas columns corresponding to CLR are shown in light grey. Within each normalisation method, columns are divided into S1 (standard untrimmed analysis, black), S2 (intersection of both standard and trimmed analysis, grey), and S3 (trimmed analysis, light grey). The heatmap colour scale represents the percentage of marker genes detected per cell type in each category, ranging from 0% (white) to 100% (red). Numbers in parentheses indicate the total number of marker genes identified in the full and trimmed datasets, respectively.

In the PBMC dataset, when 2000 features and 10 PCs were used, we compared the sets of selected markers when using the full datasets and with those obtained after removing the most extreme cells per cell type cluster. Extreme cells were defined as the 10% of cells per cell type with the largest minimum distances to their 30 nearest neighbours in the normalized gene expression space. Figures 1A and B show the t-SNE plot for the PBMC dataset, with cells classified as extreme based on *k*-nearest neighbour distances highlighted by a black outline. Furthermore, a heatmap (Fig. 1C) shows the percentages P_1_, P_2_, and P_3_ for each cell type, comparing marker sets obtained from the full data and after trimming extreme cells, under LogNormalize and CLR normalisation.

For the largest clusters, representing monocytes, NK cells, T cells, and B cells, proportions P2 are high under both LogNormalize and CLR normalisation. Under LogNormalize, P2 values are at least 87% for all four major cell types in the dataset, reaching 92% for monocytes, 89% for B cells, 88% for T cells, and 87% for NK cells. Proportions P2 for these cell types under CLR normalisation range from 81% to 86%. These results indicate that marker selection for frequent cell types in the PBMC dataset is largely robust to the removal of extreme cells, with only a small fraction of genes being specific to either the full or trimmed set. Platelets yield P2 values of 70% under LogNormalize and 58% under CLR normalisation. The overlap between markers found in both, full and trimmed datasets, was 77.5% under LogNormalize and 57% under CLR normalisation for pre-B cells (CD4-), while a lower overlap is observed for pro-B cells (CD4+), with P2 values of 40.5% and 37.3%, respectively. In both progenitor populations, larger fractions of markers are either lost after trimming (P1) or newly selected in the trimmed dataset (P3), reflecting increased sensitivity of marker selection to the presence of outlier cells.

The heatmap can also be used to compare the influence of using either LogNormalize or CLR. For monocytes, 92% of marker genes are shared between the full and trimmed data under LogNormalize, compared to 86% under CLR. Common myeloid progenitors (CMPs) show the same trend, with an overlap of 68% under LogNormalize and 51% under CLR normalisation. Higher overlap under LogNormalize is also evident for B cells, with P2 values of 89% under LogNormalize and 86% under CLR. Across cell types, LogNormalize normalisation is associated with higher overlap between marker sets from the full and trimmed data.

**Figure 2:**
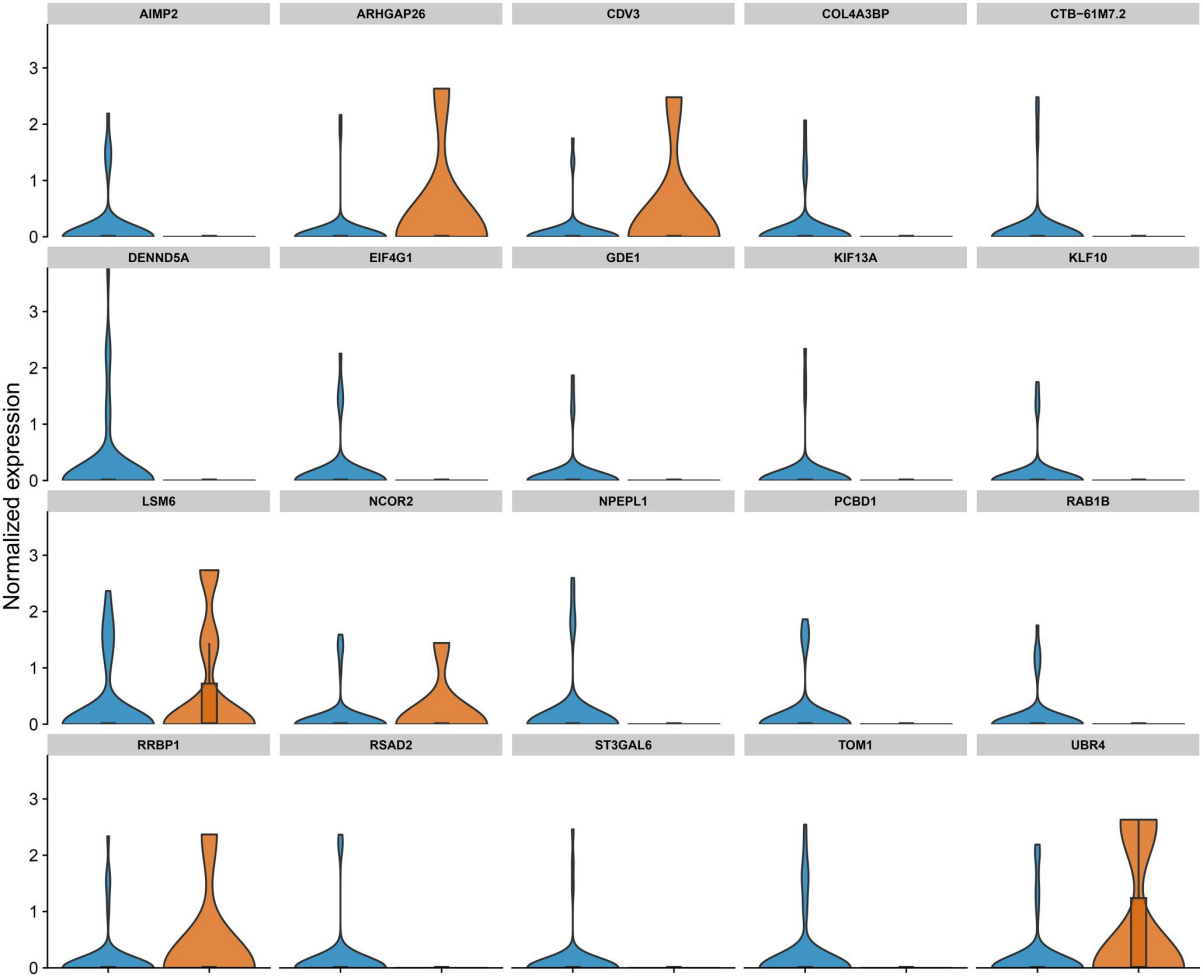
Distribution of normalised expression levels for the top 20 marker genes in pre-B cells identified in the PBMC dataset, comparing core (non-outlier) cells (blue) with the 10% most outlying cells (orange). Pre-B cells were identified as having low robustness by the scTrimClust approach. Accordingly, expression levels differ strongly between core cells and outlying cells in this cluster.

**Figure 3:**
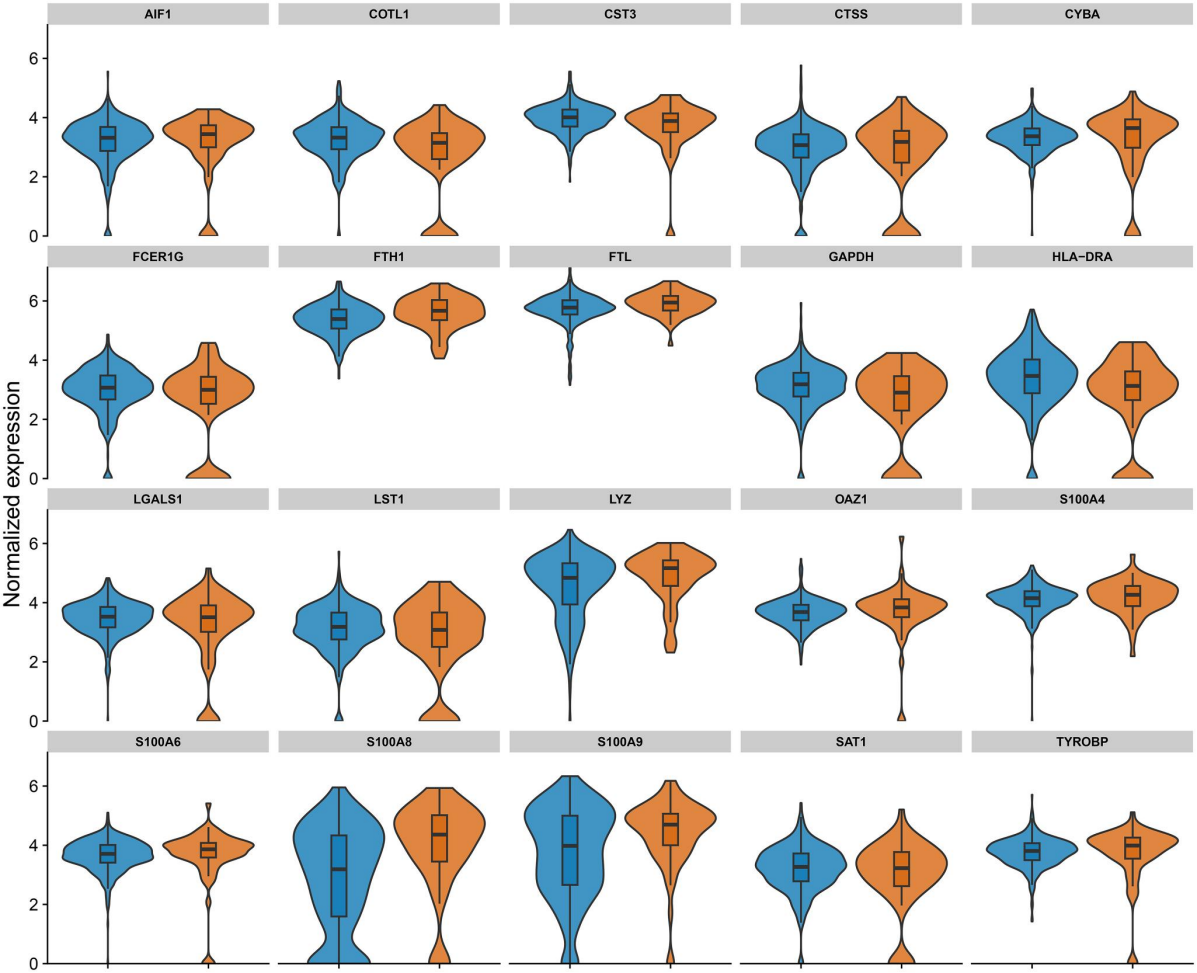
Distribution of normalised expression levels for the top 20 marker genes in monocytes identified with the PBMC dataset, comparing core (non-outlying) cells (blue) with the top 10% most outlying cells (orange). Monocytes were identified as highly robust by the scTrimClust approach. Accordingly, expression levels do not much differ between core cells and outlying cells in this cluster.

To further study the effect of extreme cells, we exemplarily studied the distribution of the top 20 marker genes in the core cells and the extreme cells for one robust cluster (monocytes) and for a cluster with low robustness (Pre-B cells). For the Pre-B cells, larger differences in the distribution can be observed (Fig. 2), while only small differences can be seen for the monocytes (Fig. 3). The same can be observed for two examples from the COVID-19 dataset (Supplementary Figs 3 and 4).

**Figure 4:**
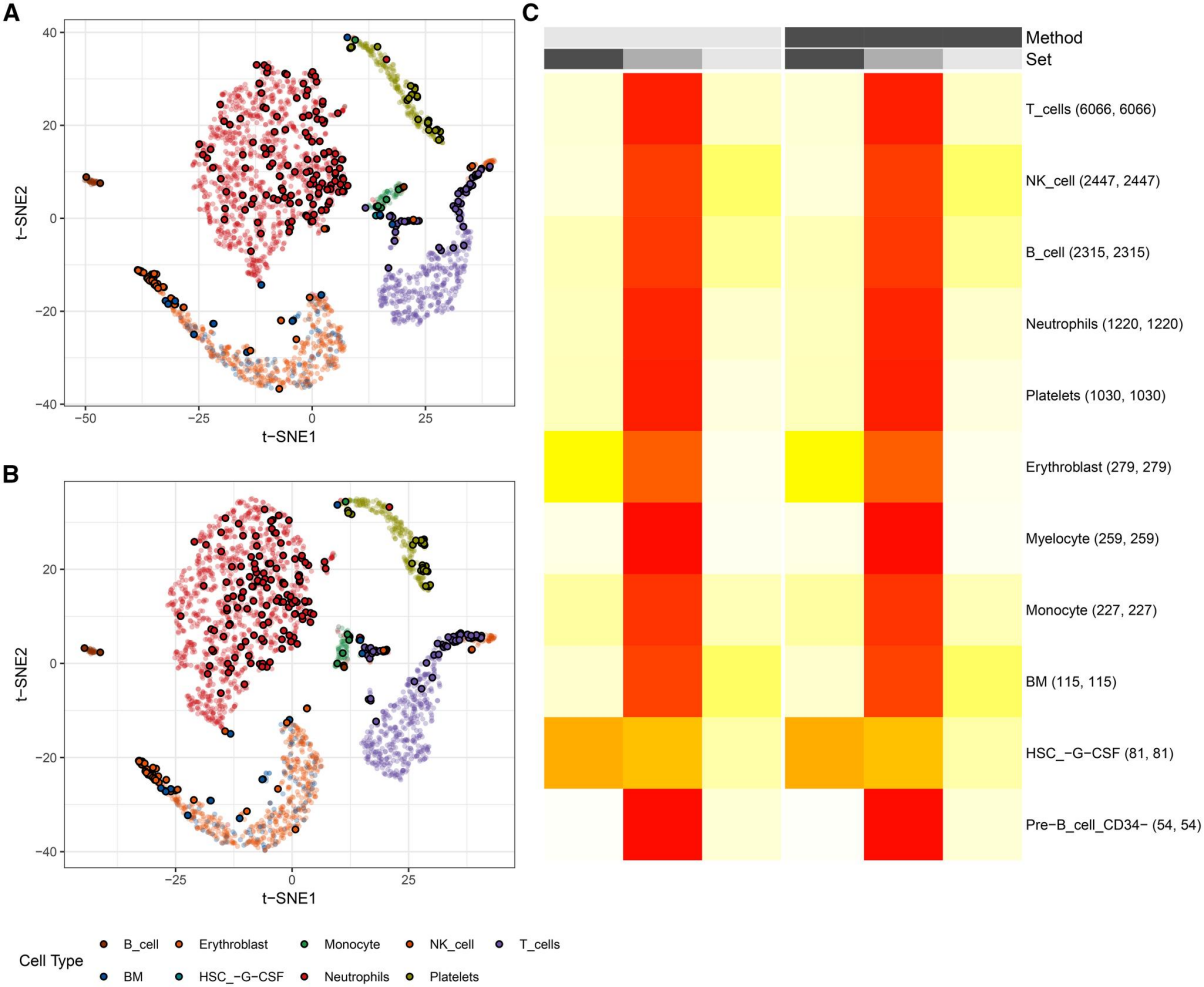
(A, B) t-distributed Stochastic Neighborhood Embedding (t-SNE) plot of the COVID-19 dataset generated using 2000 and 1000 preselected features (Both normalised via Seurat’s LogNormalize with 5 principal components selected), respectively, with cells identified as outliers (top 10% based on intracluster k-nearest neighbour distances) highlighted by a black outline. Colours represent annotated cell types. (C) Heatmap comparing results when using either 1000 or 2000 preselected genes. Columns for nFeature = 2000 are shown in black, whereas columns for nFeature = 1000 are shown in light grey. Within each feature-selection setting, columns are divided into S1 (non-trimmed analysis, black), S2 (intersection of trimmed and non-trimmed dataset, grey), and S3 (marker genes found only in trimmed dataset, light grey). Heatmap colours indicate the percentage of marker genes detected per cell type in each category, ranging from 0% (white) to 100% (red). Numbers in parentheses indicate the total number of marker genes identified in the full and trimmed datasets, respectively.

In the COVID-19 dataset, we examined the effect of trimming 10% of cells on marker robustness using LogNormalize with 5 PCs, comparing 2000 and 1000 variable features for analysis. T cells, pre-B cells (CD4-), and myelocytes have the highest overlap between marker sets from the two selection settings, with 92%, 96%, and 96% of markers shared, respectively (Fig. 4). High overlap is also observed for platelets and neutrophils, with 91% and 90% of marker genes in common between full and trimmed datasets.

NK cells, B cells, monocytes, and bone marrow cells show slightly lower but still high overlap, each around 80%–85%. Full and trimmed datasets share 72% of marker genes for erythroblasts between feature selection settings. The lowest overlap is detected in hematopoietic stem cells (HSCs), where only 43% of markers are shared. Overall, reducing the number of variable features from 2000 to 1000 does not alter the robustness of marker selection under the selected trimming strategy, with notable sensitivity primarily confined to rare cell type populations.

### 3.2 Breakdown point analysis

**Figure 5:**
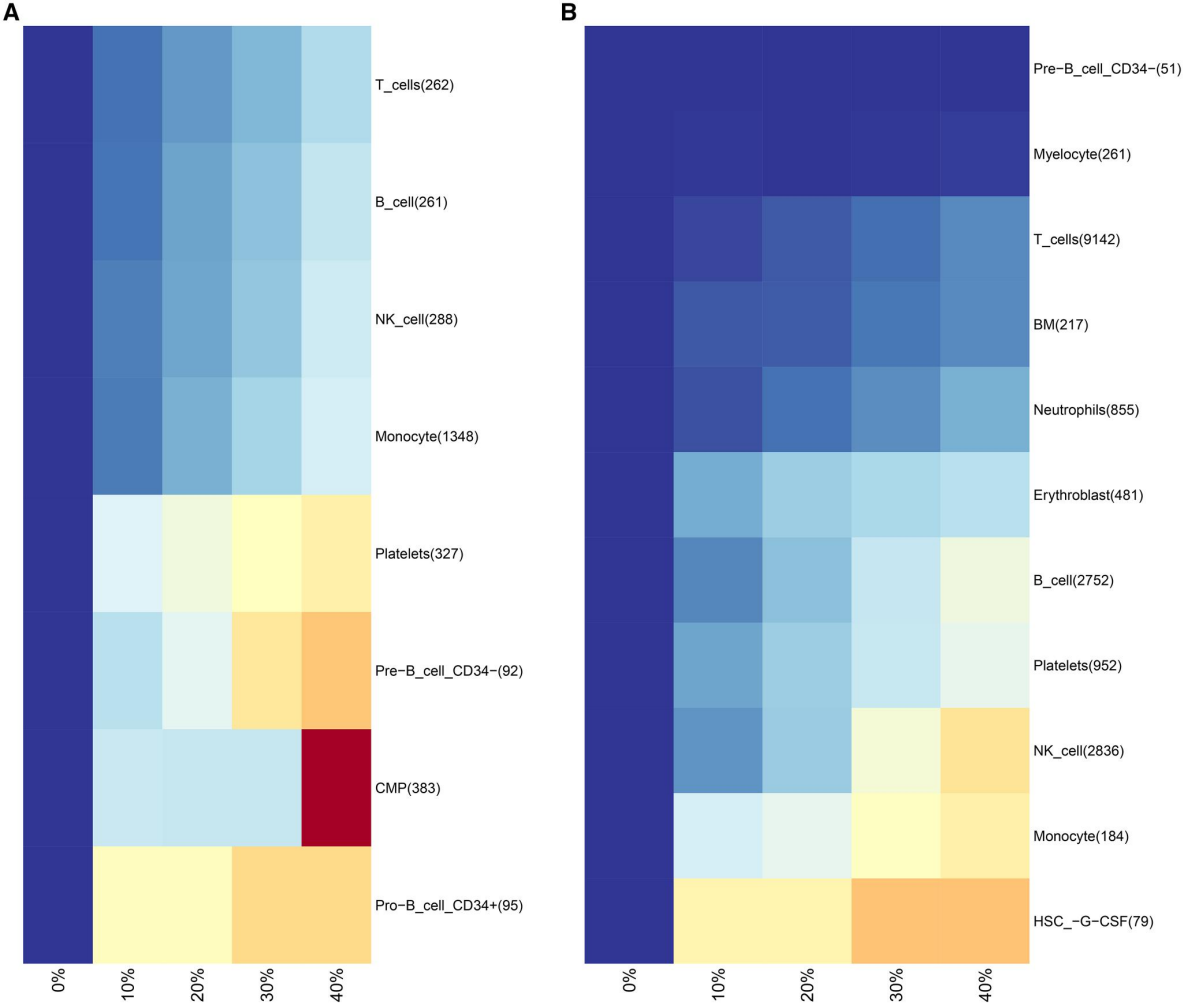
(A) Breakdown point heatmap of PBMCs (centered log ratio (CLR)-normalised, 5 principal components, 1000 variable features). (B) COVID-19 dataset under identical parameters, with colour gradient showing percentage of retained marker genes (red: 0% to blue: 100%) after trimming at 0%, 10%, 20%, 30%, and 40% thresholds, with y-axis labelling cell type clusters.

We performed a breakdown point analysis of marker selection stability across trimming levels up to 40%. Across the COVID-19 and the PBMC datasets, analysis with CLR normalisation, five principal components, and 1000 variable genes shows that the largest decrease in marker retention occurs between no trimming (0%) and the 10% trimming threshold, with more gradual changes observed at higher trimming levels (Fig. 5A). In the PBMC dataset, increasing trimming progressively reduces marker retention across most major immune cell types. Monocytes retain approximately 89% of their marker genes at 10% trimming, decreasing steadily to 62% at 40%. T cells and B cells display similar trends, retaining around 90% of markers at 10% trimming and declining to 69% and 66%, respectively, at 40%. Marker retention for NK cells decreases from 88% at 10% to 64% at 40% trimming. Platelets are characterized by lower overall stability, losing 40% of marker genes already at 10% trimming level. Progenitor populations differ in their responses to increased trimming. CD4+ pro-B cells preserve half of their marker genes on initial trimming, with percentages remaining constant, and only decreasing after 30% trimming to about 39%. Pre-B cells (CD4-) undergo continuous reduction in marker representation, from 67% at 10% trimming to 35% at 40%. Lastly, CMPs maintain a constant 65% of marker genes through 30% trimming and lose all detectable genes at 40%.

The COVID-19 dataset reveals high marker stability for T cells, with more than 97% of marker genes present at 10% trimming and over 86% remaining at 40% (Fig. 5B). Neutrophils, myelocytes, and bone marrow (BM) cells likewise maintain higher marker percentages, exceeding 85% across all trimming levels. Marker percentages for platelets and erythroblasts decrease to about 58 and 68%, respectively, at the 40% trimming threshold. With increasing trimming, marker percentages for NK cells and B cells decrease from approximately 84 and 87% to 41 and 56% at 40%. The most pronounced early loss occurs for HSCs, where marker genes fall below 50% at 10% trimming level and fall to approximately 34% at the highest trimming level. In contrast, pre-B cells (CD4-) preserve complete marker representation across all trimming levels.

### 3.3 Simulations on locational shifts of cells by dimension reduction

In the earlier version of the work (Ruff and Jung 2025), we used a different approach to identify extreme cells per cluster. This approach used concave hulls around each cell cluster on the level of the 2-D t-SNE coordinates. Cells close to the border of the hull were identified as extremes of their cluster. During the reviewing process, we became aware that the approach on the t-SNE level might not adequately mirror the distribution of cell clusters in the original high-dimensional data space due to locational shifts of individual cells. Therefore, we switched to the current approach of identifying extreme cells directly in the high-dimensional space as detailed in section 2.3.

While Chari and Pachter (2023) have also studied locational shifts of individual cells through dimension reduction methods like t-SNE or UMAP, we ran some additional simulations with artificial data under controlled settings which extend the findings of Chari and Pachter, who used real-world data for their research. In a first scenario A, we generated data for 1000 cells in the 3-D space using the normal distribution with high covariance between the three dimensions. Cells that are located far away from the centre of the distribution in the 3-D space became located in the border area of the 2-D space of t-SNE coordinates, however not evenly around the cluster (supplementary Fig. 5). With smaller covariances, extreme cells became more evenly distributed around the cell cluster in the 2-D t-SNE space (supplementary Fig. 6). Next (scenario B), we generated data again for 1000 cells within a 100-D space using either an autoregressive covariance matrix or one with a block structure. Here, also most of the cells which were far distant from their high-dimensional centre were located evenly in the border area of the cell cluster in the 2-D t-SNE space. However, individual extreme cells were now also located somewhere other than in the border area (supplementary Figs 7 and 8).

## 4 Implementation in R

We added the approach of scTrimClust in the form of four new functions to our R-package RepeatedHighDim (https://cran.r-project.org/web/packages/RepeatedHighDim/index.html). Here, we present these four functions with their main arguments. A more detailed description of how to use the functions is given in the supplementary material.

### 4.1 Identification of cells in the border area

Both hull-based and distance-based robustness procedures described here are available in the R package RepeatedHighDim (available from version 2.5.0 onward). The distance-based approach is provided through the function scTrimDist. A Seurat object is used as input, and trimming is performed within user-defined cell type or clustering labels. Here, kNN distances in gene expression space are used to identify peripheral cells, which are removed as extreme cells to assess robustness. Neighbourhood size is controlled by knn_k, which determines how many nearby cells contribute to each distance calculation and thereby shapes how local information is aggregated when identifying peripheral observations. The overall extent of trimming is governed by keep_frac (ranging from 0 to 1), which specifies the fraction of cells retained per cell type or per cluster. A modified t-SNE plot highlights excluded cells, and a trimmed, processed Seurat object is provided together with cluster specific marker genes and kNN result.

scTrimClust visualizes cell clusters in a low-dimensional space (t-SNE, UMAP, etc.) and removes cells from the border area of each cluster. The hull.alpha parameter controls the concavity of cluster boundaries using an alpha hull. Higher values produce smoother, more inclusive hulls, while lower values create tighter, more irregular contours. The outlier.quantile parameter sets the percentile cutoff (0–1) for minimum cell-to-hull distances, classifying cells below this threshold as extremes. Lower values restrict detection to extreme outliers, whereas higher values identify more peripheral cells as outliers.

The output includes a modified plot with flagged or removed outlier cells, along with the hull coordinates required to generate cluster boundaries, a list of ahull objects for each cluster, and the coordinates for both non-outlier and outlier cells. It also returns the Seurat object, with outlier cells removed if remove.outliers=TRUE. The processed Seurat object enables seamless integration of scTrimClust into existing Seurat workflows, where DimPlot would typically be used, allowing users to continue downstream analyses without outliers.

### 4.2 Comparative visualization of marker selection

The scTC_trim_effect function quantifies changes in cluster-specific marker genes after outlier removal by comparing untrimmed (default Seurat) and trimmed (scTrimClust-processed) datasets. It takes a list of marker genes produced by Seurat’s FindAllMarkers function. The output is a heatmap displaying cell clusters (rows) and three categories per method (columns): markers exclusive to untrimmed data, shared markers, and markers exclusive to trimmed data. An example is given in Figs 1 and 4. Colours represent the percentage of markers in each category (0%–100%).

While scTC_trim_effect compares marker sets across methods at a fixed trimming level, scTC_bpplot evaluates marker retention across trimming percentages for a single method. Trimming percentages are specified via the outlier.quantile parameter in scTrimClust. The heatmap displays cell clusters (rows, labelled with original marker counts) and trimming percentages, using a colour gradient (default: 0% = red, 100% = blue) to show the percentage of original markers retained. High retention (colder colours) indicates robust clusters, while warmer colours reflect marker loss after trimming. An example is given in Fig. 5.

## 5 Discussion

Advances in sequencing technology provide deeper insights into molecular mechanisms, however, as larger datasets arise from these experiments, more uncertainty is introduced as well. Uncertainty can come from the complex bioinformatics pipelines for omics analyses which consist of many steps where the analyst must draw decisions about parameter settings in nearly each step. Here, we focus on the uncertainties in the data itself by trying to identify cell specimens which may not perfectly represent a particular cell type. This contrasts with time-consuming resampling approaches which put each single observation on the scale. Though bootstrap and other resampling approaches have a long tradition to evaluate the robustness of findings in omics data (Pepe *et al.* 2003, Saremi *et al.* 2021, Hemandhar Kumar *et al.* 2024), they treat each observation to the same degree and can be very time-inefficient for large datasets and complex analysis pipelines. By focusing on the identification of extreme observations, we follow the concept of trimmed statistics. To evaluate the robustness of a finding, the analyst must run the whole analysis only twice: once for the full dataset and once for the trimmed one. Hubert *et al.* (2005) also used the idea of downweighing outliers in transcriptomics data when doing PCA.

Our proposed scTrimClust method can be useful for different purposes. First, and more generally, robust and less robust results can be distinguished by comparing results from trimmed and full data sets. Additionally, the analyst can use scTrimClust to study how robust different steps or parameters of the analysis (such as normalisation or selection of number of preselected features) are in the presence of outlying cells.

We have demonstrated that our approach is also useful to compare the robustness between two variants of the analysis, e.g. two different normalisation methods or two different approaches in the clustering step. Sun *et al.* (2019) have also shown how sensitive scRNA-seq is to the choice of the clustering method. Therefore, by trimming cells with potential wrong cluster membership, we can study the effect of the extreme or outlying cells of a cluster.

As most computational methods, scTrimClust has some limitations, which we are currently working on to diminish. Detection of extreme data points is not straightforward, and based on different metrics or distributional assumptions, different approaches can identify different outlying cells. As demonstrated by simulations in section 3.3, outlier detection in dimension reduced data appears mostly inappropriate compared to outlier detection in the original high-dimensional space, because locational structures may not be preserved by dimension reduction, even with t-SNE approaches improved for preservation of locations (Zhou and Sharpee 2022). Nevertheless, even when different methods for outlier detection can identify different cells, our proposed approach is still useful to compare the robustness of selected markers sets, since it does not depend on the specific methods applied here. Despite uncertainties in outlier detection, we have demonstrated that scTrimClust can distinguish between robust and non-robust markers. This was very evident when comparing the distributions of gene expression levels between core and outlying cells for robust and non-robust markers.

A larger limitation is the application of our approach to less frequent cell types. Trimming cells from a small population decreases statistical power and may lead to a higher false negative rate.

In summary, while other work (Yuan *et al.* 2022; Liu *et al.* 2023) has been proposed to obtain robust results in scRNA-seq analysis, little research has been done to study the sensitivity of marker selection in the presence of outlying cells. With our work, we propose a first approach to account for this issue. Furthermore, our approach is more targeted than standard bootstrap analyses, which would select from all cells with the same weight.

## Supplementary Material

vbag082_Supplementary_Data

## Data Availability

RepeatedHighDim is available as an R package at https://cran.r-project.org/web/packages/RepeatedHighDim/index.html. The PBMC 3k dataset used in this work is available at https://cf.10xgenomics.com/samples/cell/pbmc3k/pbmc3k_filtered_gene_bc_matrices.tar.gz. The COVID-19 dataset, originally generated by Silvin *et al.* (2020), is available from the ArrayExpress Archive of Functional Genomics Data under accession number E-MTAB-9221: https://www.ebi.ac.uk/biostudies/arrayexpress/studies/E-MTAB-9221.
