# Supplementary material for: scTrimClust: a fast approach to robust scRNA-seq analysis using trimmed cell clusters
Source: Bioinform Adv. 2026 Mar 16;6(1):vbag082. doi: 10.1093/bioadv/vbag082 (PMC13105839; doi:10.1093/bioadv/vbag082)
Supplement: vbag082_Supplementary_Data [file vbag082_supplementary_data.pdf]

scTrimClust: A Fast Approach to Robust scRNA-seq Analysis Using Trimmed Cell Clusters

Sergej Ruff[1], Klaus Jung[1]*

1) Institute of Animal Genomics, University of Veterinary Medicine Hannover, Hannover, Germany

* Correspondence: klaus.jung@tiho-hannover.de

Supplementary Material

A) Supplementary Figures

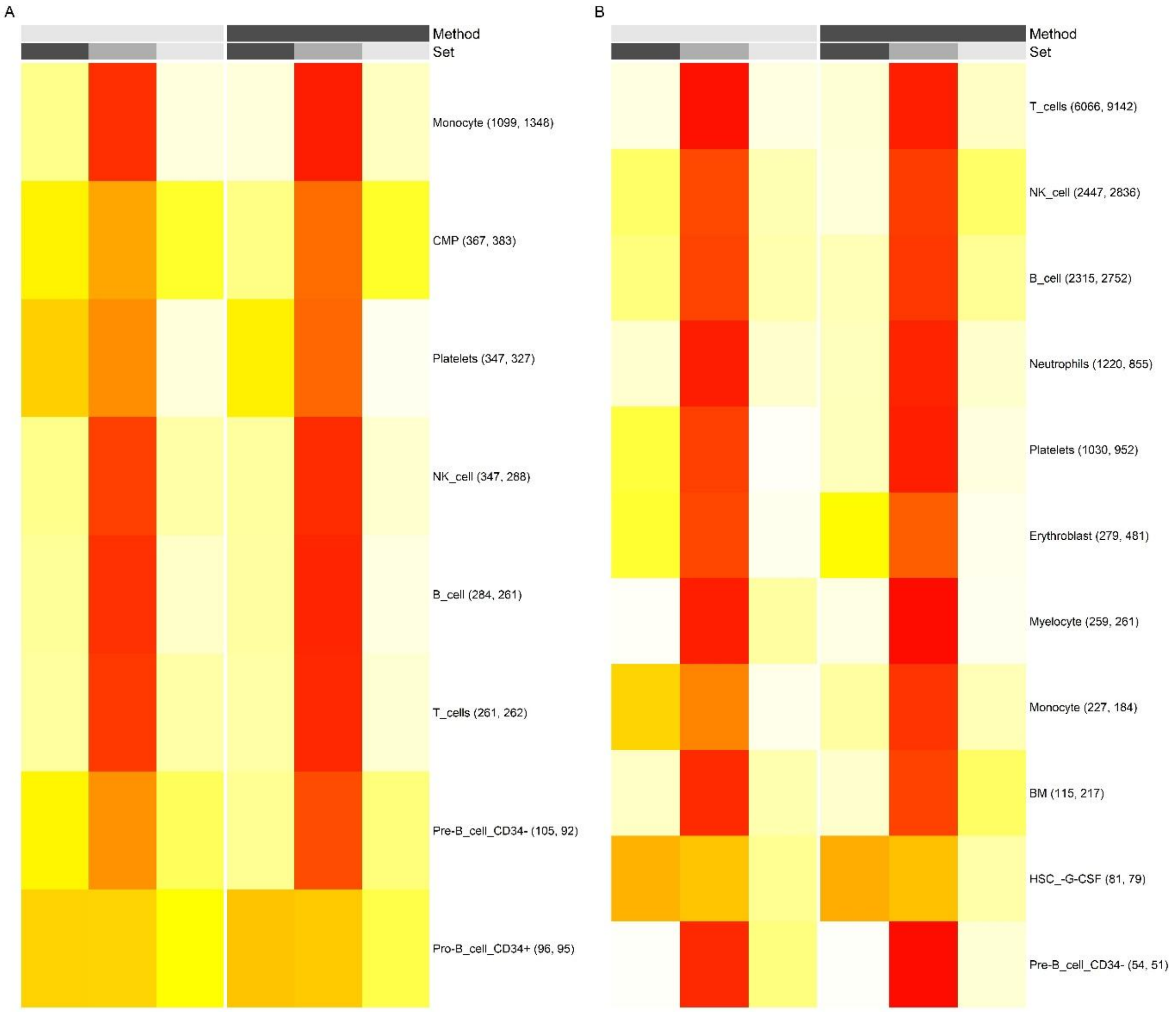

Supplementary Figure 1. (A) Heatmap comparing percentage of marker genes in full and trimmed PBMC dataset after CLR and LogNormalize (LogNorm) normalisation with five principal components and 1,000 variable features. (B) Heatmap comparing percentage of marker genes in full and trimmed COVID dataset after CLR and LogNorm normalisation with five principal components and 1,000 variable features. Columns for LogNorm are shown in black, whereas columns for CLR are shown in light grey. Within each normalisation-selection setting, columns are divided into S1 (non-trimmed analysis, black), S2 (intersection of trimmed and non-trimmed dataset, grey), and S3 (marker genes found only in trimmed dataset, light grey). Heatmap colours indicate the percentage of marker genes detected per cell type in each category, ranging from 0% (white) to 100% (red). Numbers in parentheses indicate the total number of marker genes identified in the full and trimmed datasets, respectively

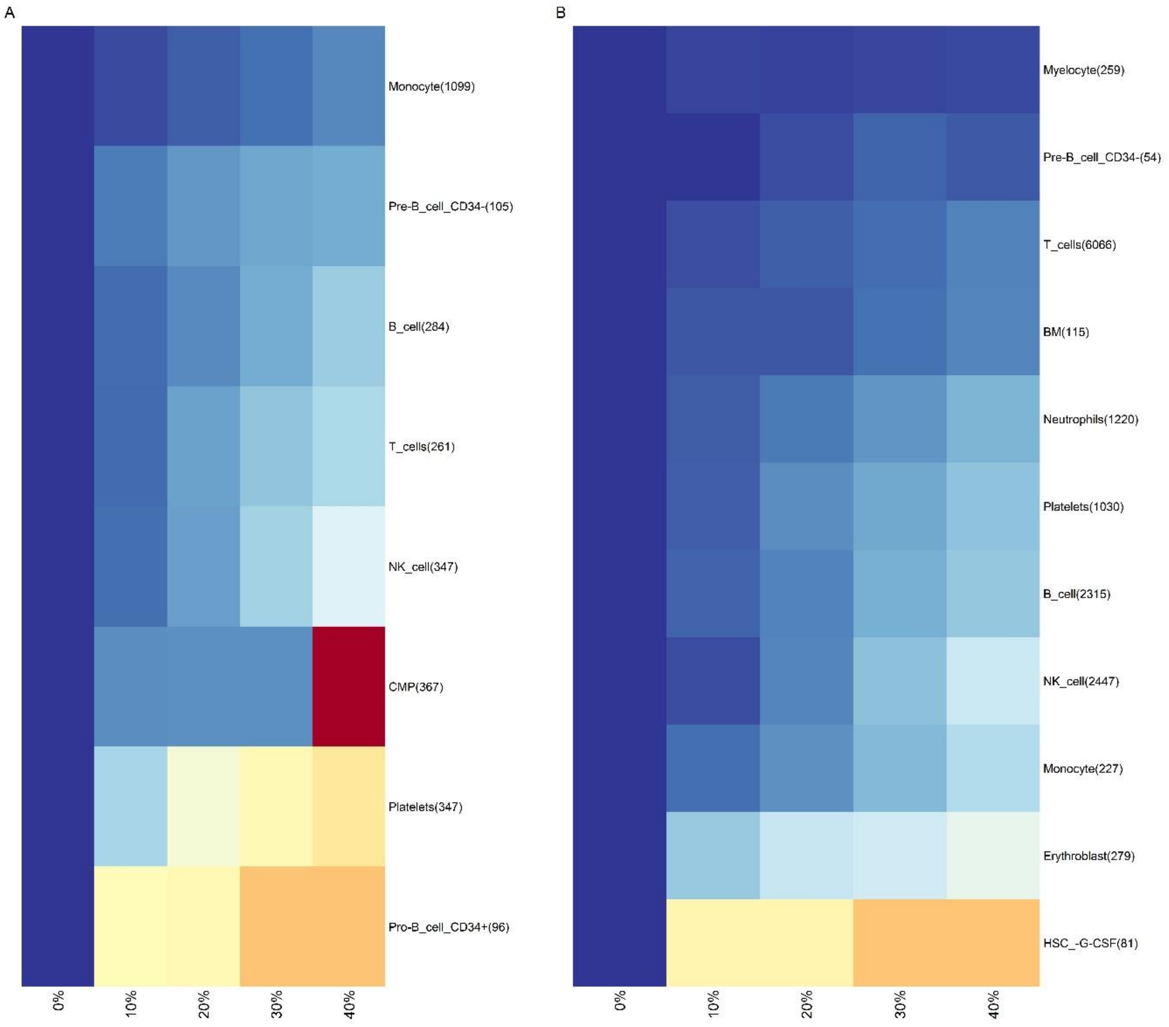

Supplementary Figure 2. (A) Breakdown point heatmap of PBMCs (LogNormalize (LogNorm) normalisation, 5 principal components, 1,000 features). (B) COVID dataset under identical parameters, with colour gradient showing percentage of retained marker genes (red:0% to blue:100%) after trimming at 0%, 10%, 20%, 30%, and 40% thresholds, with y-axis labelling cell type clusters

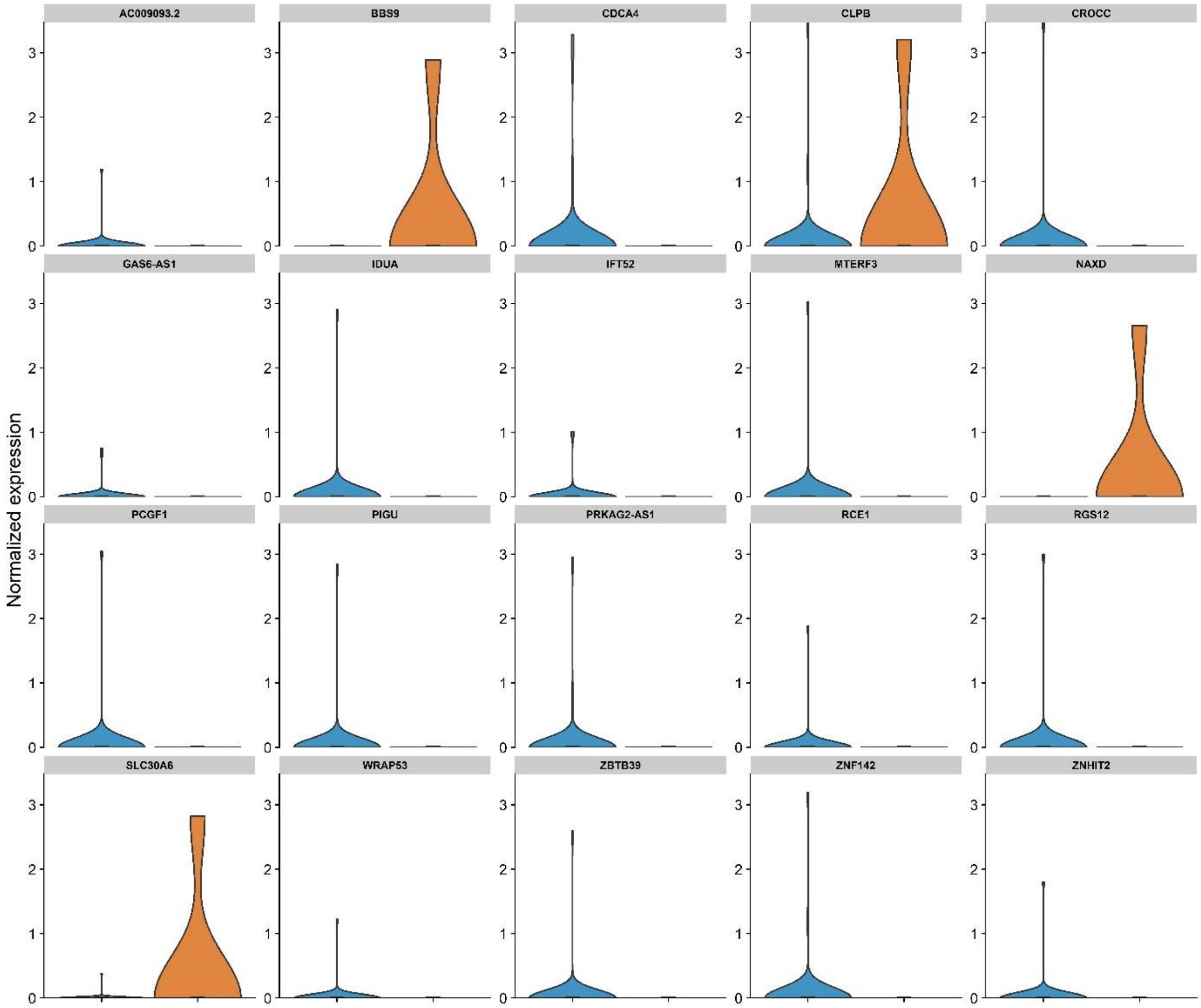

Supplementary Figure 3. Distribution of normalised expression levels for the top 20 marker genes in monocytes identified with the COVID dataset, comparing core (non-outlying) cells (blue) with the top 10% most outlying cells (orange).

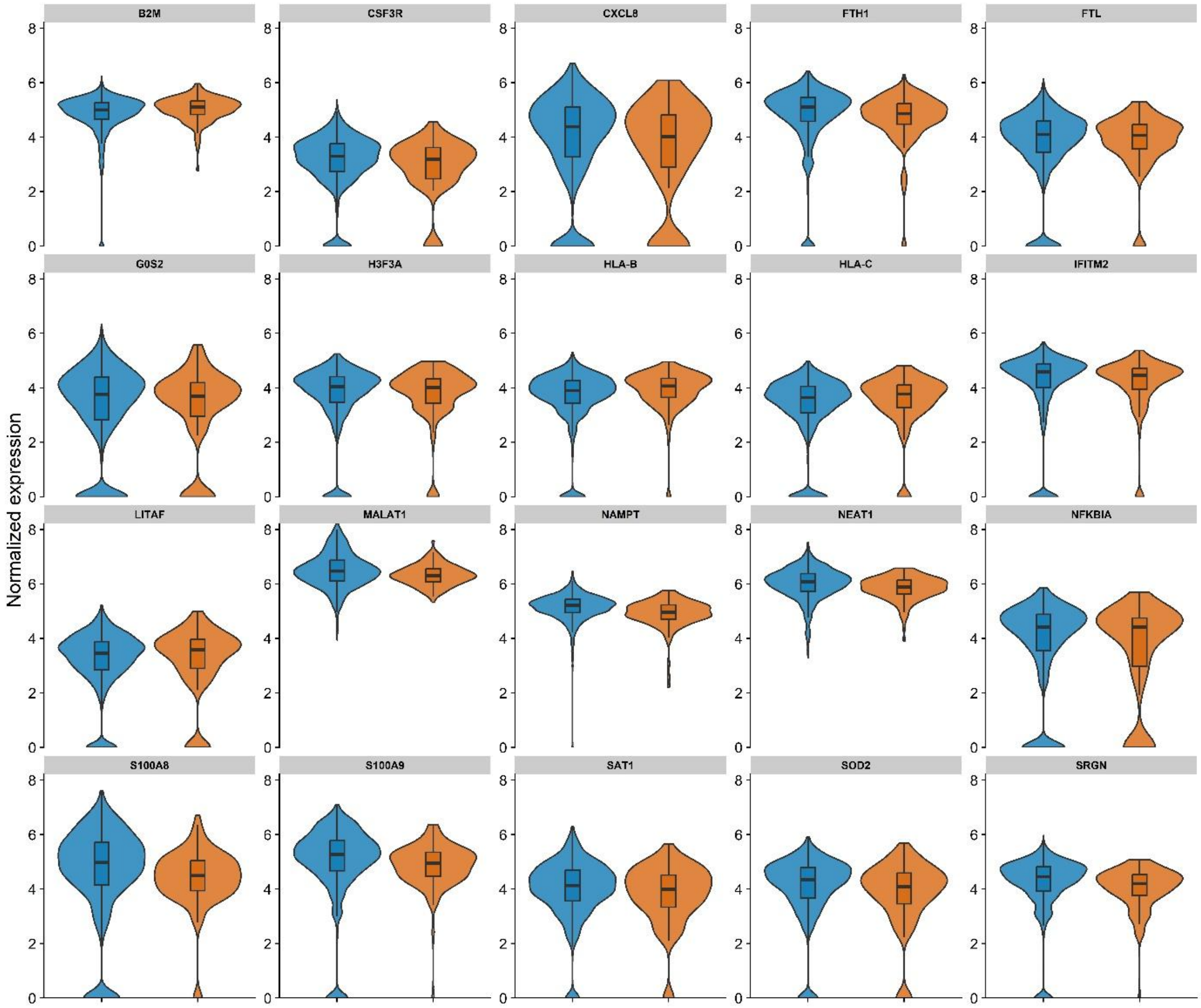

Supplementary Figure 4: Distribution of normalised expression levels for the top 20 marker genes in neutrophils identified with the COVID dataset, comparing core (non-outlying) cells (blue) with the top 10% most outlying cells (orange).

$$\mathcal{N}_3(\mu, \Sigma) \text{ with } \mu = \mathbf{0} \text{ and } \Sigma = \begin{pmatrix} 1 & 0.9 & 0.9 \\ 0.9 & 1 & 0.9 \\ 0.9 & 0.9 & 1 \end{pmatrix}$$

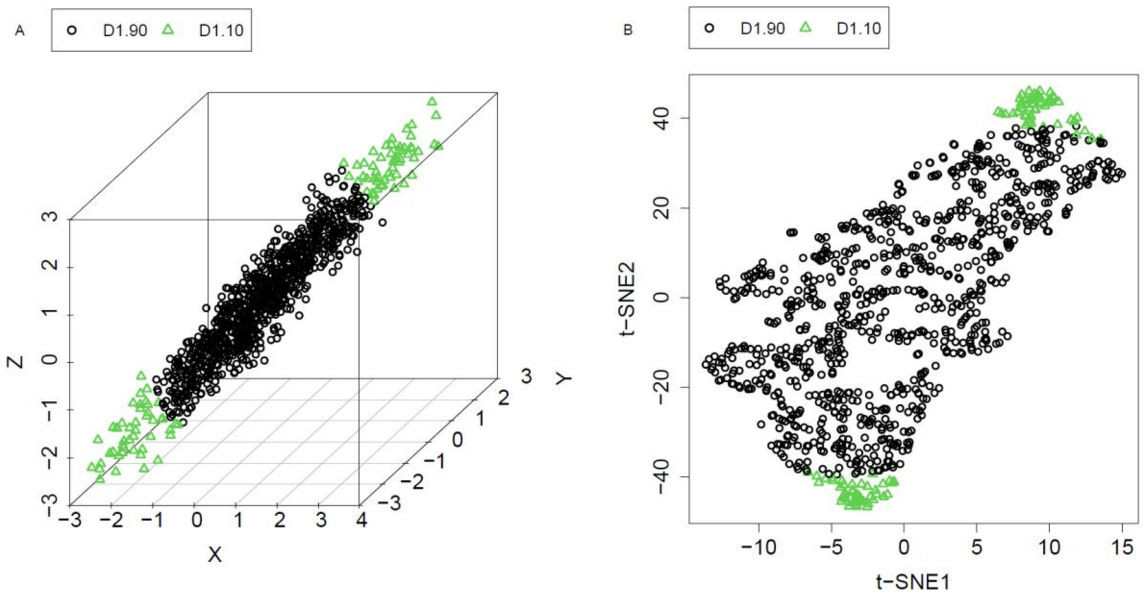

Supplementary Figure 5. (A) Data for n=1,000 cells, generated by the 3-dimensional normal distribution, with large covariances between the dimensions. 10% of cells with the largest distance from the centre of the distribution are labelled in green. (B) The same data after dimension reduction with t-SNE. The extreme cells are in the border area of the cluster but not evenly distributed around this area.

$$\mathcal{N}_3(\mu, \Sigma) \text{ with } \mu = \mathbf{0} \text{ and } \Sigma = \begin{pmatrix} 1 & 0.9 & 0.5 \\ 0.9 & 1 & 0.1 \\ 0.5 & 0.1 & 1 \end{pmatrix}$$

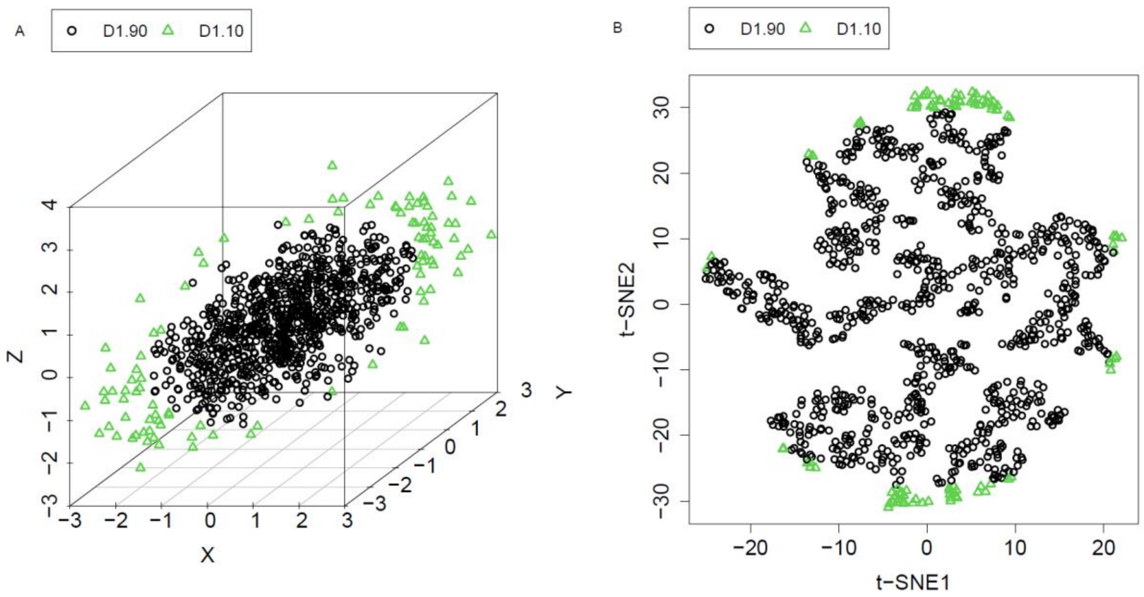

Supplementary Figure 6. (A) Data for n=1,000 cells, generated by the 3-dimensional normal distribution, with large to small covariances between the dimensions. 10% of cells with the largest distance from the centre of the distribution are labelled in green. (B) The same data after dimension reduction with t-SNE. The extreme cells are in the border area of the cluster and a bit more evenly distributed around this area.

$\mathcal{N}_{100}(\mu, \Sigma)$ with $\mu = \mathbf{0}$ and $\Sigma = 0.95^{|i-j|}$

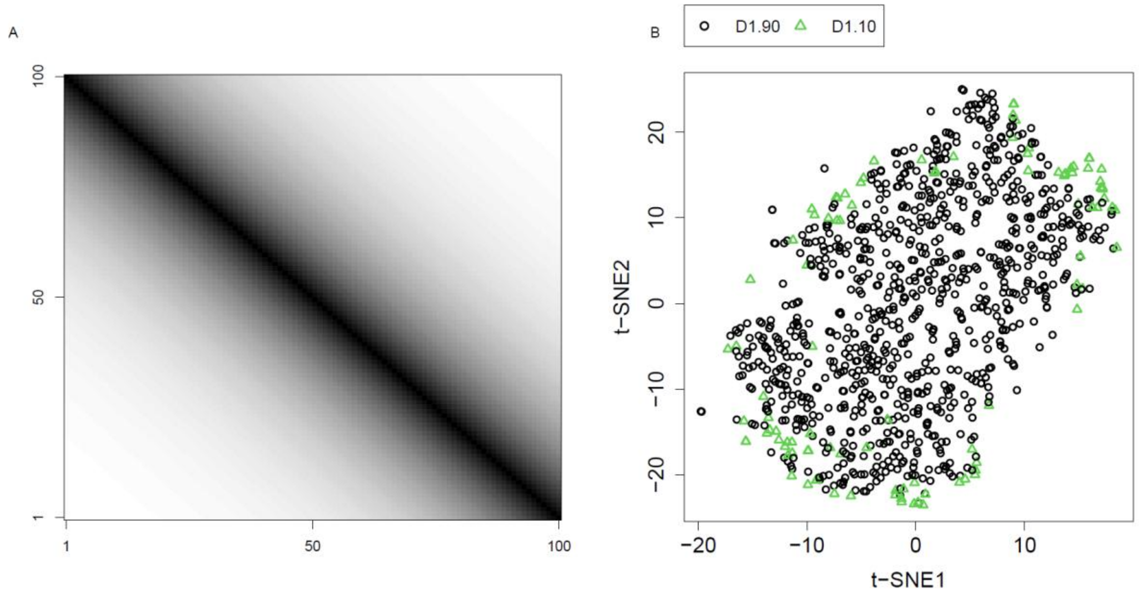

Supplementary Figure 7. Data for n=1,000 cells, generated by the 100-dimensional normal distribution, with autoregressive covariance matrix. (A) Visualization of the covariance matrix. (B) Data after dimension reduction with t-SNE. 10% of cells with the largest distance from the high-dimensional centre of the distribution are labelled in green. The extreme cells are located evenly in the border area of the cluster, but a few of these extreme cells are also placed not in the border area.

$$\mathcal{N}_{100}(\mu, \Sigma) \text{ with } \mu = \mathbf{0} \text{ and } \Sigma = \begin{pmatrix} 0.9 & 0.0 & 0.0 & 0.0 & 0.0 \\ 0.0 & 0.7 & 0.0 & 0.0 & 0.0 \\ 0.0 & 0.0 & 0.5 & 0.0 & 0.0 \\ 0.0 & 0.0 & 0.0 & 0.3 & 0.0 \\ 0.0 & 0.0 & 0.0 & 0.0 & 0.1 \end{pmatrix} \otimes \mathbb{J}_{m/5}$$

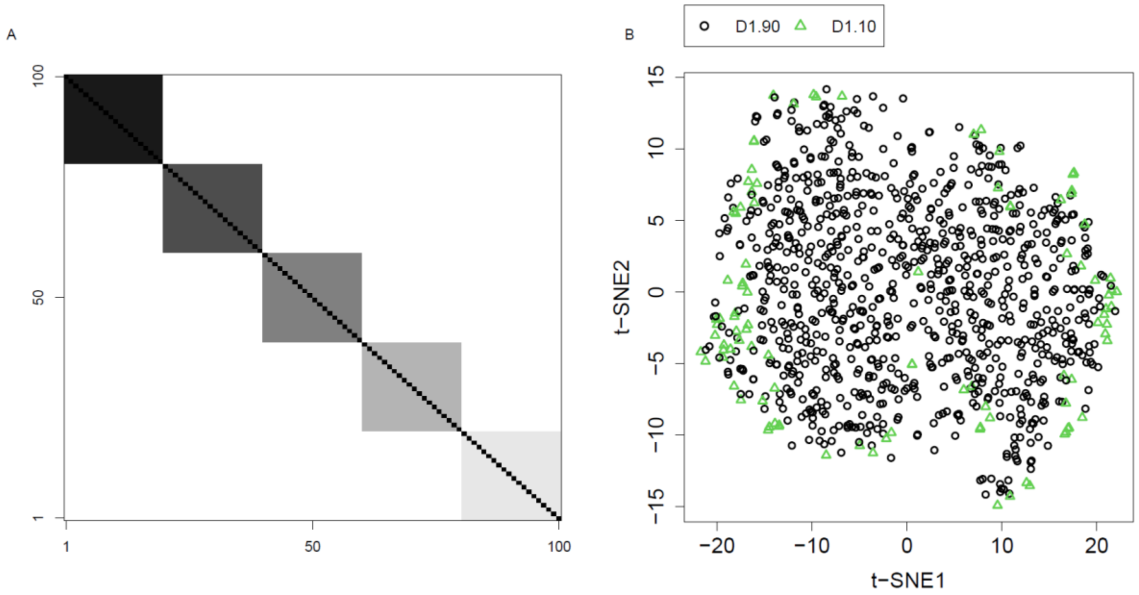

Supplementary Figure 8. Data for n=1,000 cells, generated by the 100-dimensional normal distribution, with autoregressive covariance matrix. (A) Visualization of the covariance matrix. (B) Data after dimension reduction with t-SNE. 10% of cells with the largest distance from the high-dimensional centre of the distribution are labelled in green. The extreme cells are located evenly in the border area of the cluster, but some of these extreme cells are also placed not in the border area.

B) R-Vignette for scTrimClust

Preprocessing of input data

The identification of outlier cells for each cell type based on cluster hulls requires a preprocessed Seurat object containing a low-dimensional embedding (e.g., t-SNE or UMAP). We demonstrate this using the 2,700 Peripheral Blood Mononuclear Cell (PBMC) data set from the Seurat guided clustering tutorial (https://satijalab.org/seurat/articles/pbmc3k_tutorial.html), publicly available from 10x Genomics (https://cf.10xgenomics.com/samples/cell/pbmc3k/pbmc3k_filtered_gene_bc_matrices.tar.gz).

In short, the following preprocessing steps must be completed before using scTrimClust (here demonstrating with t-SNE as the dimensionality reduction method):

```
library(Seurat)
library(magrittr)

pbmc_sc <- Read10X("data_original/blooddata_hg19/") %>%
  CreateSeuratObject(project = "pbmc3k", min.cells = 3, min.features = 200)
%>%
  {PercentageFeatureSet(., pattern = "^MT-") -> .[["percent.mt"]]; .} %>%
  subset(.,subset = nFeature_RNA > 200 & nFeature_RNA < 2500 & percent.mt <
5) %>%
  NormalizeData() %>%
  FindVariableFeatures(nfeatures = 2000) %>%
  ScaleData() %>%
  RunPCA(npcs = 10) %>%
  FindNeighbors(dims = 1:10) %>%
  FindClusters(resolution = 0.5) %>%
  RunTSNE(dims = 1:10)
```

The process includes quality control filtering, data normalization, variable feature selection, principal component analysis (PCA), and t-SNE dimensionality reduction. The preprocessed pbmc_sc seurat object contains the following components:

```
pbmc_sc
An object of class Seurat
13714 features across 2638 samples within 1 assay
Active assay: RNA (13714 features, 2000 variable features)
 3 layers present: counts, data, scale.data
 2 dimensional reductions calculated: pca, tsne
```

Gene expression profiles for 13,714 genes across 2,638 cells are stored in pbmc_sc, with 2,000 variable features within the RNA assay, three data layers (counts, data, scale.data), and computed PCA and t-SNE dimensionality reductions. Cell type annotation can be

performed manually or be automated using the SingleR package, which relies on reference expression data such as the Human Primary Cell Atlas (HPCA). The HPCA data set is included in the celldex R package.

```
library(SingleR)
library(celldex)

pbmcsce <- as.SingleCellExperiment(pbmc_sc)
pbmcse <- as(pbmcsce, "SummarizedExperiment")
rownames(pbmcse) <- rownames(pbmcsce)
ref <- HumanPrimaryCellAtlasData()
pbmc_sc$CellAnnotation <- SingleR(
  test = pbmcse,
  ref = ref,
  labels = ref$label.main
)$labels
```

Here, cell type annotations are stored in the 'CellAnnotation' metadata column for use within the scTrimClust function. Alternatively, the function can utilize numeric cluster IDs from the 'seurat_clusters' column (output by FindClusters) to identify cluster-specific outliers.

Identifying outlier cells with scTrimClust

We added the approach of scTrimClust in form of four new functions to our R-package RepeatedHighDim (https://cran.r-project.org/web/packages/RepeatedHighDim/index.html). scTrimClust identifies and removes outlier cells within user-defined clusters, operating on a preprocessed Seurat object containing cluster annotations (e.g., the pbmc_sc data set with labels stored in the 'CellAnnotation' metadata field). To maintain compatibility with Seurat, we use the package's native DimPlot function to visualize cells during outlier detection, displaying them on dimension reduction embeddings such as UMAP, t-SNE, or PCA. scTrimClust inherits all parameters from the DimPlot function and adds new ones specific to it.

```
scTCoutput <- scTrimClust(pbmc_sc,
                       reduction = 'tsne',
                       group.by = 'CellAnnotation',
                       add.alpha.hull = TRUE,
                       hull.alpha = 2,
                       remove.outliers = FALSE,
                       outlier.quantile = 0.1,
                       outlier.alpha = 0.2)
```

The hull.alpha parameter controls the concavity of cluster boundaries using a convex hull, implemented via the ahull function from the alphahull package. Higher values produce smoother, more inclusive hulls, while lower values create tighter, more irregular contours. The outlier.quantile parameter sets the percentile cutoff (0-1) for minimum cell-

to-hull distances, classifying cells below this threshold as outliers. Lower values restrict detection to extreme outliers, whereas higher values identify more peripheral cells as outliers.

Additional parameters allow further customization: add.alpha.hull allows the hull around each cluster to be added or removed, remove.outliers excludes outlier cells from both the plot and the returned Seurat object, and outlier.alpha adjusts the transparency of outlier cells when remove.outliers = FALSE. Unlike DimPlot, which returns only a ggplot object, scTrimClust returns a list of objects.

```
print(names(scTCoutput))

[1] "plot"                "object"
[3] "nonoutliers_coords"  "outlier_coords"
[5] "d_ahull_coords"      "ahull_list"
```

The output includes a modified plot with flagged or removed outlier cells, along with the hull coordinates required to generate cluster boundaries (d_ahull_coords), a list of ahull objects for each cluster (ahull_list), and the coordinates for both non-outlier (nonoutlier_coords) and outlier cells (outlier_coords). It also returns the Seurat object (object), with outlier cells removed if remove.outliers = TRUE. The processed Seurat object enables seamless integration of scTrimClust into existing Seurat workflows, where DimPlot would typically be used, allowing users to continue downstream analyses without outliers.

<u>Identifying outlier cells with scTrimDist</u>

In addition to the hull-based trimming strategy implemented in scTrimClust, we provide a complementary distance-based approach implemented in the function scTrimDist. While scTrimClust relies on geometric cluster boundaries in the low-dimensional embedding, scTrimDist operates directly on high-dimensional normalized gene expression space, making it independent of the choice of visualization method.

```
dist_res <- scTrimDist(
    seurat_obj = pbmc_sc,
    celltype_col = 'CellAnnotation',
    knn_k = 30,
    keep_frac = 0.90,
)
```

Here, seurat_obj is the same processed Seurat object with processed cell type labels used by scTrimClust. The argument celltype_col specifies the metadata column that defines the cell types or clusters within which outlier detection is performed. In this example, cell types assigned by SingleR are stored in 'CellAnnotation' are used. The parameter knn_k defines the number of nearest neighbors used to compute local neighborhood distances in the normalized gene expression space. The number of cells

being trimmed is controlled by keep_frac, which defines the number of cells to keep. A value of 0.9 means that 10% of cells within each cell type should be trimmed. The output of scTrimDist is a named list. It includes a visualization of detected outliers (plot_outliers). A trimmed Seurat object (trimmed_object) is returned for further downstream analysis. Lastly, the output contains a table of cluster-specific marker genes (all_markers) identified after trimming.

Compare the effect of trim on marker genes for different methods

The scTC_trim_effect function quantifies changes in cluster-specific marker genes after outlier removal by comparing untrimmed (default Seurat) and trimmed (scTrimClust-processed) data sets. Assume we have already identified outliers using scTrimClust (Section 4.2), generating the trimmed Seurat object scTCoutput$object. Both pbmc_sc (untrimmed) and scTCoutput$object (trimmed) contain cell type annotations in the 'CellAnnotation' metadata column (Section 4.1), which we set as active identities for marker detection.

```
# Set CellAnnotation as active identity for both data sets
Idents(pbmc_sc) <- "CellAnnotation"          # Untrimmed
Idents(scTCoutput$object) <- "CellAnnotation"   # Trimmed
```

Marker gene lists from both untrimmed and trimmed data sets are required. These are generated using the FindAllMarkers function from Seurat.

```
markers_untrimmed <- FindAllMarkers(pbmc_sc)
markers_trimmed <- FindAllMarkers(scTCoutput$object)
```

Marker genes must be paired as lists comparing untrimmed and trimmed results. Colors for methods and gene set categories (original, shared, trimmed-exclusive) are user-defined.

```
method_pairs <- list(
  Default = list(data1 = markers_untrimmed, data2 = markers_trimmed)
)
method_colors <- c(Default = "#2CA02C")   # method color (green)
set_colors <- c(
  "S1:standard" = "#4D4D4D",    # Untrimmed-exclusive markers
  "S2:intersect" = "#AEAEAE",   # Shared markers
  "S3:trimmed" = "#E6E6E6"      # Trimmed-exclusive markers
)
```

The output is a heatmap displaying cell clusters (rows) and three categories per method (columns): markers exclusive to untrimmed data, shared markers, and markers exclusive to trimmed data. Colors represent the percentage of markers in each category (0-100%).

```
scTC_trim_effect(
  method_pairs = method_pairs,
  method_colors = method_colors,
```

```
  set_colors = set_colors,
  column_title = "PBMC Data set: Marker Changes After Trimming"
)
```

Trimming and breakdown point analysis

While scTC_trim_effect compares marker sets *across methods* (e.g., CLR vs. LogNormalize (LogNorm)) at a fixed trimming level, scTC_bpplot evaluates marker retention across trimming percentages for a single method. Trimming percentages are specified via the outlier.quantile parameter in scTrimClust. Below, we analyze the PBMC data set trimmed at 10%, 20%, 30%, and 40% (using the same normalization method, e.g., LogNorm,nfeatures = 2,000, nPCs = 10), comparing marker retention to the original untrimmed (0%) data set.

We assume that the steps described in section 4.2 were already performed for 0%, 10%, 20%, 30%, and 40% trimmed. We then obtain the marker genes for each trimming level via FindAllMarkers.

```
pbmc_0trim <- FindAllMarkers(pbmc_sc)
pbmc_10trim <- FindAllMarkers(pbmc_10trimmed_object)
pbmc_20trim <- FindAllMarkers(pbmc_20trimmed_object)
pbmc_30trim <- FindAllMarkers(pbmc_30trimmed_object)
pbmc_40trim <- FindAllMarkers(pbmc_40trimmed_object)
```

We then use all marker gene lists as input for scTC_bpplot.

```
scTC_bpplot(
  pbmc_0trim, pbmc_10trim, pbmc_20trim, pbmc_30trim, pbmc_40trim,
  trim_percent_vector = c(0, 10, 20, 30, 40),
  legend_title = "Log-Normalized PBMC: Marker Retention Across Trimming
Levels")
```

The heatmap displays cell clusters (rows, labeled with original marker counts) and trimming percentages (columns, e.g., 0%, 10%, 20%,30% and 40%), using a color gradient (default: 0% = red, 100% = blue) to show the percentage of original markers retained. High retention (warmer colors) indicates robust clusters, while cooler colors reflect marker loss after trimming.
